# Norwegian e-Infrastructure for Life Sciences (NeLS)

**DOI:** 10.12688/f1000research.15119.1

**Published:** 2018-06-29

**Authors:** Kidane M. Tekle, Sveinung Gundersen, Kjetil Klepper, Lars Ailo Bongo, Inge Alexander Raknes, Xiaxi Li, Wei Zhang, Christian Andreetta, Teshome Dagne Mulugeta, Matúš Kalaš, Morten B. Rye, Erik Hjerde, Jeevan Karloss Antony Samy, Ghislain Fornous, Abdulrahman Azab, Dag Inge Våge, Eivind Hovig, Nils Peder Willassen, Finn Drabløs, Ståle Nygård, Kjell Petersen, Inge Jonassen

**Affiliations:** 1 Computational Biology Unit, Department of Informatics, University of Bergen, Bergen, Norway; 2 University of Oslo, Oslo, Norway; 3 Department of Clinical and Molecular Medicine, Norwegian University of Science and Technology, Trondheim, Norway; 4 University of Tromsø - The Arctic University of Norway, Tromsø, Norway; 5 Department of Animal and Aquacultural Sciences, Faculty of Biosciences, Norwegian University of Life Sciences, Ås, Norway

**Keywords:** Data management and sharing, compute and storage infrastructure, microservices, federated authentication, integration API, Galaxy, ELIXIR Norway

## Abstract

The Norwegian e-Infrastructure for Life Sciences (NeLS) has been developed by ELIXIR Norway to provide its users with a system enabling data storage, sharing, and analysis in a project-oriented fashion. The system is available through easy-to-use web interfaces, including the Galaxy workbench for data analysis and workflow execution. Users confident with a command-line interface and programming may also access it through Secure Shell (SSH) and application programming interfaces (APIs).

NeLS has been in production since 2015, with training and support provided by the help desk of ELIXIR Norway. Through collaboration with NorSeq, the national consortium for high-throughput sequencing, an integrated service is offered so that sequencing data generated in a research project is provided to the involved researchers through NeLS. Sensitive data, such as individual genomic sequencing data, are handled using the TSD (Services for Sensitive Data) platform provided by Sigma2 and the University of Oslo. NeLS integrates national e-infrastructure storage and computing resources, and is also integrated with the SEEK platform in order to store large data files produced by experiments described in SEEK.

In this article, we outline the architecture of NeLS and discuss possible directions for further development.

## 1. Introduction

The Norwegian ELIXIR node is coordinated by the University of Bergen (UiB) and comprises the University of Oslo (UiO), The Arctic University of Norway (UiT), the Norwegian University of Science and Technology (NTNU), and the Norwegian University of Life Sciences (NMBU). The node provides services, training, and support to a broad range of national users, largely life-science researchers and students ^1^. These scientists usually work in collaborative projects and need to store, analyze, and share data sets, often large in size, throughout all stages of the project, and between various platforms and computational resources. However, many of these users do not feel comfortable using a command-line interface, and have limited programming, system administration, or data management skills.

Commercial workbenches such as the BaseSpace Sequence Hub ^a^ and Geneious ^b^ aim at user accessibility, but offer computation and data sharing only within their closed and expensive platform setups. On the other hand, the open-source SEEK serves as a platform for sharing systems biology project data, transparently and for free ^2, 3^. Notably, the integrative open-source GenomeSpace enables organizing and sharing data not only between users, but also between various workbenches and computational resources ^4– 6^. Although powerful, its setup could not be adapted to integrate with the available and required e-infrastructure resources in Norway.

The Norwegian e-Infrastructure for Life Sciences (NeLS) was built upon the previous experiences with developing and using bioinformatics workbenches in Norway, for example: the Genomic HyperBrowser ^7– 9^, an extension of Galaxy ^10, 11^; the easy-to-use UiO Bioportal ^12^, later replaced by a Galaxy-based Lifeportal ^13^; or eSysbio, a workbench prototype for data sharing and systems biology workflows ^c^. NeLS provides users in Norway and their collaborators abroad an integrated system for data storage and sharing, as well as data processing and analysis. NeLS allows users to efficiently and safely store, analyze, and share their genomics-scale data and analyses, all through the use of web interfaces. Most Norwegian users can log in using the credentials – user name and password – they use at their home institution, other users need to register.

**Figure 1.**
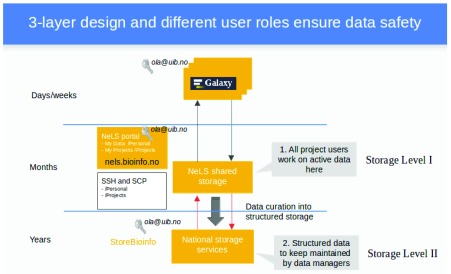
Overview over short-term and long-term storage in NeLS.

NeLS has a three-layered architecture ( Figure 1). The intermediate layer (Storage Level I) provides data storage intended to be used by projects in an active analysis phase (with data being kept in this storage layer for months). Data can be accessed (and up- and downloaded) through a web portal, as well as through Secure Shell (SSH) and application programming interfaces (APIs). The latter two provide command-line-confident users with a more efficient way to work with data. The top level constitutes the data analysis workbench of NeLS. For this, we have chosen the popular Galaxy, an open-source, web-based workbench for accessible, reproducible, and transparent computational omics. Galaxy allows computational workflows to be set up and used without the need of programming skills. Our Galaxy instances have limited storage capacity and it is therefore intended that data resides on this level only for short periods of time, in the range of weeks. The bottom layer (Storage Level II) offers long-term storage, provided by the National Infrastructure for Research Data (NIRD), a generic e-infrastructure operated by Sigma2 ^d^. Here, data can be stored for years, requiring a more strictly organized structure with defined types and metadata.

NeLS includes Galaxy instances hosted at the five universities constituting the Norwegian ELIXIR node. These instances have a basic catalogue of tools and workflows that are relevant for researchers in life sciences, as well as more specialized ones depending on the focus of the hosting institution. NeLS provides tools integrated into Galaxy to easily push and pull data from the persistent Storage Level I. Some of the Galaxy servers are integrated with high-performance computing (HPC) resources – provided by Sigma2 – for transparent execution of computationally intensive tools.

In this article, we describe the architecture of this integrated e-infrastructure and examples of its usage, and outline the possible directions of future developments.

## 2. The architecture of NeLS

**Figure 2.**
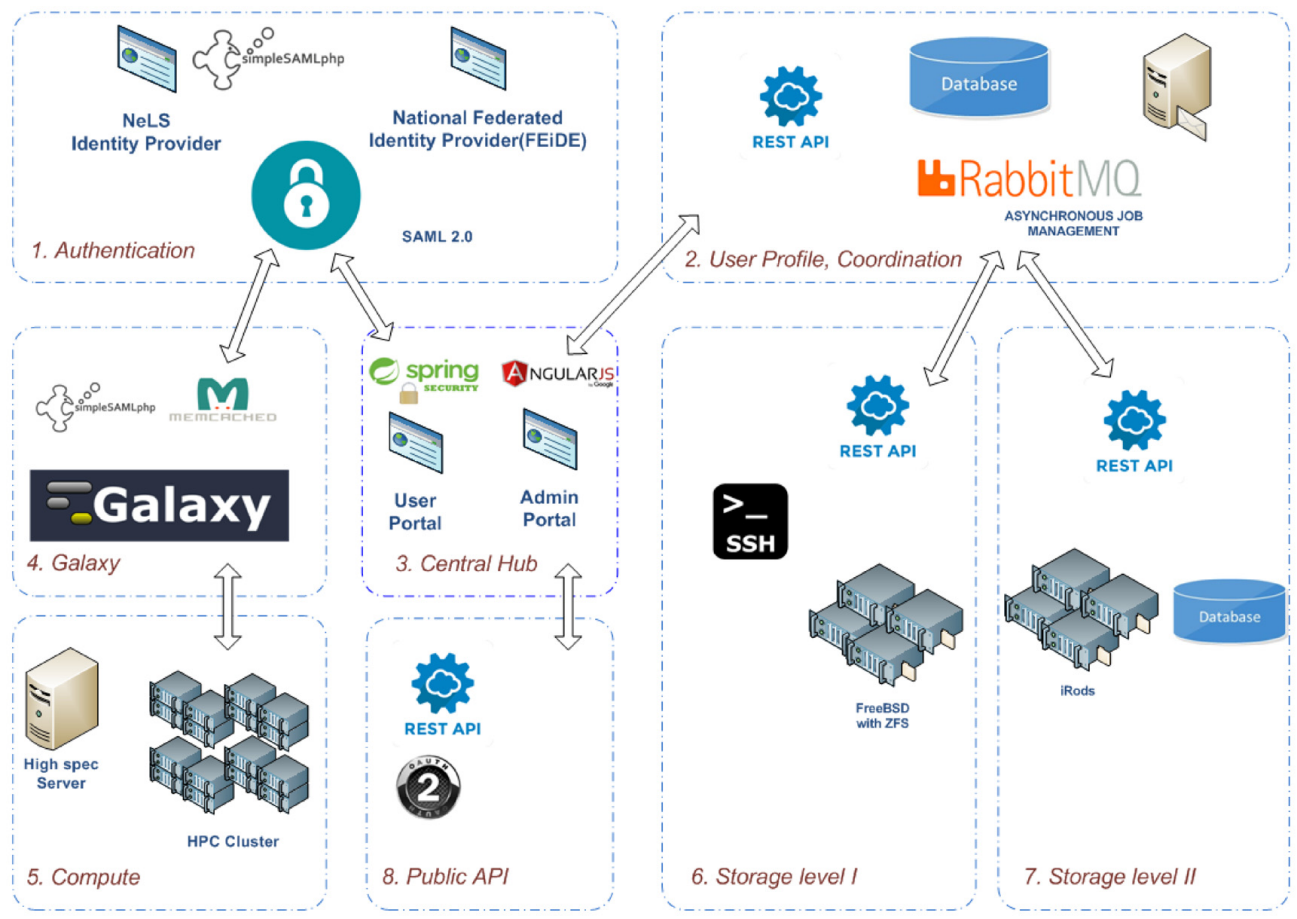
NeLS Architecture.

The Norwegian e-Infrastructure for Life Sciences was not built as a top-down, grand design and implementation exercise. Rather, it was implemented through time by focusing on different parts of the problem at a time and always striving to make a functional whole. It was decided early on to avoid re-inventing the wheel and rather base the system on proven solutions and practices whenever possible. In addition, addressing different concerns in isolation while keeping the big picture in mind has proven to be an effective way for constructing the NeLS system. In the end, there were many components of different flavors: off-the-shelf systems, adaptations of available open-source packages, and also custom in-house developed systems. Figure 2 shows a componentized architecture of the NeLS system. In the following subsections, we describe the components of the NeLS system.

### 2.1 Authentication

Authentication is a process by which the user’s credentials are verified against a user-information catalogue in order to determine whether the user is who they claim to be, before granting access to resources. NeLS supports multiple identity sources based on the Security Assertion Markup Language 2.0 (SAML 2.0) standard ^e^. Currently, it supports the Norwegian Federated Electronic Identity service (FEiDE ^f^) and NeLS’s own identity provider. NeLS’s identity provider was constructed by configuring Simple-SAMLphp ^g^, an open-source security software system. Integration with the ELIXIR Authentication and Authorisation Infrastructure (AAI) ^15^ as an identity provider has been tested technically, and can in the future be used to differentiate resource allocations further in the NeLS network of services.

### 2.2 User-profile management and coordination

The first time a user logs in using any of the supported identity providers, NeLS creates a user profile and subsequent derived identities. Secure Shell (SSH) access credentials are generated for the user and can be fetched from the NeLS portal (central hub). This coordination layer holds metadata about projects and associated membership of users. The user-profile management and coordination block provides a Representational State Transfer (REST, "RESTful") web application programming interface (API) ^16– 18^ for other units, and enables asynchronous job management by leveraging an off-the-shelf message-queuing system, RabbitMQ ^h^. Structured logging, e-mail communication, and related management tasks are supported in this block.

### 2.3 The NeLS portal (central hub)

The NeLS portal is the central hub of the whole system. It is a Java-based web application with multiple responsibilities and uses the Spring Security package ^i^ to interface with the different SAML 2.0 identity providers. Upon first login, NeLS creates a profile for the user and initializes all necessary components. Following are the four distinct responsibilities of the NeLS portal:

(a) Web-based file-system browser to the NeLS Storage Level I (see subsection 2.6).
(b) Initiate and monitor asynchronous jobs for copying, moving, and sharing files within the Level I storage layer, as well as transfer across storage Levels I and II.
(c) Facilitate OAuth ^j^ token provision. The NeLS portal acts as a bridge towards the identity providers and avails NeLS metadata for the OAuth service (see subsection 2.4).
(d) Interface with external systems. The NeLS portal has been successfully integrated with a national solution for sensitive data, the TSD ^k19^. NeLS makes two-factor authentication of the TSD easier for users, and provides an easy web-based way for initiating data-transfer jobs between the NeLS and TSD infrastructures.

### 2.4 Galaxy

The Galaxy block is the workhorse of the NeLS system. It gives the user a curated set of tools and workflows supported by the ELIXIR Norway help desk ^1^. Technically, the Galaxy block comprises Galaxy in a remote-user configuration, and an authentication layer in front to interface with the same set of identity providers as the NeLS portal. SimpleSAMLphp in service provider (SP) configuration with its AuthMemCookie ^l^ solution on top of Memcached ^m^ is used to interface with the Apache web server, transforming the SAML 2.0 authorization information into a Galaxy-compatible format. NeLS also provides Galaxy tools for data import and export, which work in tandem with the NeLS portal to give the user the possibility of pulling data from the Storage Level I in NeLS into a Galaxy history, and also be able to push results of Galaxy jobs into the NeLS Storage Level I.

NeLS provides different Galaxy instances hosted by different institutions.

### 2.5 Compute block

The NeLS compute block executes NeLS Galaxy jobs. The jobs are either executed on the same high-spec (fat) servers as the Galaxy server, or they can be submitted to a high-performance computing (HPC) cluster for parallel execution. The job execution details, including HPC job management, are hidden for the user. The HPC jobs are run using a pre-allocated compute quota.

We use the Light-weight Runner (LWR, now renamed to Pulsar ^n^) Galaxy services to submit computationally intensive Galaxy jobs to an HPC system (such as the Stallo supercomputer in the UiT Galaxy ^o^). LWR communicates with Galaxy via the Galaxy API and a RabbitMQ Advanced Message Queuing Protocol (AMQP) message queue. It specifies the required parameters for the tool and executes a wrapper script for the tool. The wrapper creates temporary directories, submits tool jobs to the HPC scheduler (PBS/Torque ^p^) with selected parameters, saves results, and deletes temporary files. Once the jobs are completed, LWR transfers the data back to Galaxy for the user to inspect.

### 2.6 Storage Level I

This layer of NeLS provides flexible storage with advanced access control list to allow appropriate sharing and data protection in scientific projects.

The NeLS Storage Level I layer features a dedicated private directory for each user’s personal data as well as a project-based shared storage area for collaboration and sharing. A user can be added to a NeLS project with three possible roles: member, power-user, or principal investigator (PI). Each role has a predefined set of permissions allowed in the project area. Technically, the NeLS Storage Level I is built using FreeBSD ^q^ with its support for the ZFS ^r^ file system. It employs advanced access control lists and also provides SSH access to more tech-savvy users. It provides a RESTful web API (Java) and command-line management tools (Python).

### 2.7 Storage Level II

Level II of the NeLS storage, also known as the StoreBioinfo layer, enforces more strict organization of datasets, and is facilitated through integration with national storage resources provided by the Norwegian Infrastructure for Research Data (NIRD). Its purpose is to act as a long-term storage and data warehouse, with capacity to hold all of a project’s generated data, from raw to final results, including any data replicated to the Storage Level I. It has a metadata database and interfaces with the data-warehousing system, iRods ^s^, via specialized server-side scripts. The NeLS Storage Level II provides a RESTful web API and is orchestrated via the NeLS central coordination block (3. Central Hub in Figure 2).

### 2.8 Public API

The NeLS public API is a RESTful web service targeted towards external systems. It supports implicit and authorization-code OAuth ^t^ grant profiles. It exposes a well-defined navigation and linking mechanism into the structured data of NeLS Storage Level II. The OAuth service is an in-house built Python-based system that uses Tornado ^u^ and python-oauth2 ^v^ libraries by interfacing with the NeLS portal. In collaboration with Digital Life Norway ^w^, the public API is developed to support integration with the SEEK data management system ^2, 3^, to allow a resolvable URL to a dataset in NeLS be referenced in SEEK.

## 3. Operation

NeLS is inherently a distributed infrastructure of multiple microservices which naturally would be deployed on different servers. The scale and availability of the different resources to be integrated – such as compute, storage, identity providers, databases, *etc.* – heavily influences its deployment. In Norway, the NeLS production instance is deployed on 7 different servers, including 2 storage master systems (additional slave storage nodes – sub-systems – are not counted).

For testing or a proof-of-concept setup, all microservices and web components are possible to run on a single host with 2–4 cores and 64GB of memory, while the storage levels would naturally require their own setups.

## 4 Workflows

To cover the most prominent NeLS user needs, Galaxy workflows for analyzing RNA sequence data (prokaryotic and eukaryotic), and workflows for both the taxonomic and functional profiling of metagenomic data have been developed. In addition, workflows for the analysis of miRNAs and ChiP-seq analysis are available, see Table 1. All workflows are maintained in order to provide the state-of-the-art tools for the analysis to the users. Upon demand, each work-flow can be modified to accommodate specific user needs, *e.g.* that a tool is replaced by another tool, or version. A complete overview of the NeLS workflows and links to the Galaxy instance in which a workflow is available can be found in the NeLS portal.

**Table 1.** Current NeLS workflows

Category	Name	Description	Node
DNA-seq	Germline variant calling	Discovery of germline variation in DNA-seq samples	UiO
	Somatic variant calling	Discovery of somatic variation based on a sample pair	UiO
	LiceBase ^20^ ^z^ cDNA mapping	cDNA to genome mapping workflow for sea lice samples	UiB
RNA-seq	Eukaryote RNA-Seq	DE analysis between two collections of eukaryote samples	NTNU/UiB
	Prokaryote RNA-Seq	DE analysis between two collections of prokaryote samples	UiT
	LiceBase RNA-Seq	Alignment and count workflow for multiple Sea Lice samples	UiB
	RNA-seq counts - STAR ^21^	Create RNA count matrix from RNA-seq FASTQ files	UiB
	RNA-seq counts - HISAT2 ^22^	Create RNA count matrix from RNA-seq FASTQ files	UiB
miRNA-seq	miRNA prediction	Prediction of miRNA	UiO
	miRNA processing	Alignment and DE analysis between two collections of samples	NTNU
ChIP-Seq	ChIP-Seq analysis	*workflows in testing, to be released soon*	NTNU/UiO
Metagenomes	Taxonomic classification	Taxonomic profiling of 16S rRNA reads from shotgun reads	UiT
	META-pipe ^23, 24^	Functional annotation of assembled metagenomic shotgun samples	UiT

To ensure data reproducibility and to reduce the compute time for the user, a common data repository with pre-indexed reference genomes has been built. The repository is available across all five Galaxy instances.

For first-time users of the NeLS Galaxy, a quick start guide that contains information on the Galaxy basics is available on each NeLS Galaxy start page, and more detailed documentation and tutorials on the NeLS workflows are also available there. Finally, the user can contact the national help desk or access a Q&A forum directly from the NeLS Galaxy.

## 5 Use cases

### 5.1 Main steps in an ordinary NeLS project

**Figure 3.**
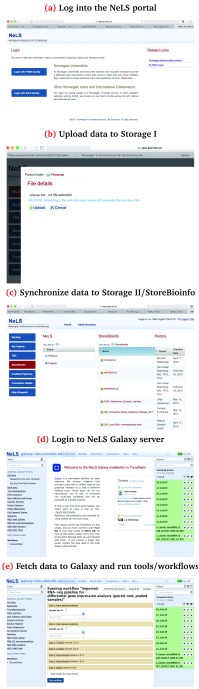
Illustration of the main stps in an ordinary NeLS project.

In a project with non-sensitive data, a user will perform the following steps (See Figure 3e (a)-(e))

(a) Log in to the NeLS portal. If the user does not have an account, and neither has a FEiDE account, they can easily apply for a NeLS account.
(b) Upload data to NeLS repository using SSH or Filezilla ^x^. If the data are generated by a Norwegian high-throughput sequencing core facility, *e.g.* one linked with the Norwegian Consortium for Sequencing and Personalized Medicine (NorSeq ^y^), the user is offered to have the data uploaded directly from the core facility.
(c) (Recommended) Synchronize data to Storage Level II (StoreBioinfo) for annotation and long-term storage.
(d) Log in to one of the Galaxy instances, *e.g.* ?https://galaxy-ntnu.bioinfo.no.
(e) Get data from NeLS Storage Level I to Galaxy, and run Galaxy tools and/or workflows. Share or publish Galaxy history.
(f) Copy new results to Storage Level I and synchronize to Storage Level II (recommended).

### 5.2 META-pipe

META-pipe ^23– 25^, developed within the ELIXIR Marine metagenomics project ^26, 27^, efficiently produces full-length annotated genes from metagenomic assemblies, and offers the extensive annotation options, flexibility, and visualization needed to pick interesting targets for further investigation. The NeLS version of META-pipe provides taxonomic and functional analysis from whole-genome shotgun sequence data. It supports high-throughput sequencing data and provides assembly, focusing the analysis on full-length genes. The pipeline consists of three major modules: preprocessing, taxonomic classification, and functional analysis. All modules are available as individual workflows, except for assembly in pre-processing, which is run manually on either a high-memory computer or our supercomputer. Workflows can be tailored to the specific needs for the analysis of a sample and it is also possible to add additional steps or to omit some of the steps.

To use META-pipe, the user follows the generic steps for login and data upload above, using the NeLS Galaxy instance of UiT ^aa^ and the NeLS portal to administrate the data of the metagenomics project. Input files are transferred from the NeLS project in Storage Level I, to the Galaxy history of the user using the provided NeLS data transfer tools in Galaxy tool menu.

To analyze the data, the user selects the META-pipe tool in Galaxy and then configures the pipeline parameters such as which tools to run, input files, reference database versions, and output formats. Once the workflow is configured, the user presses the execute button in Galaxy to execute the pipeline in the background. This will create a history element in Galaxy where the user can view the current status of the job. Currently queued or running jobs are colored yellow, and completed jobs are colored green. When the job is done, the user can examine the output data in the Galaxy view panel, transfer results to the NeLS project in Storage Level II, or download the files to their own computer.

### 5.3 Sensitive data

NeLS was not designed for hosting sensitive data such as human genome data from Norwegian patients. ELIXIR Norway is collaborating with the TSD project and infrastructure ^ab19^, created by USIT (The University Centre of Information Technology) at the University of Oslo, to offer a service to researchers in Norway for storing and processing sensitive data, including health data.

NeLS allows for seamless data transfer from the NeLS storage services (Layer I) to the TSD File Lock servers for import of supplementary non-sensitive data that user projects would need available inside TSD to interpret their sensitive data.

Workflow development for sequencing data analysis *etc.* performed in ELIXIR Norway is implemented either as tools and workflow definitions for Galaxy or as software containers, such that workflows can be deployed in the appropriate compute environment to facilitate analysis of both sensitive and non-sensitive data. ELIXIR Norway and TSD are working towards a Galaxy service in TSD, and the aspect of workflow mobility is also a key aspect in the Nordic project Tryggve2 ^ac^, with ELIXIR partners from Norway, Finland, Sweden, and Denmark, and respective national infrastructures for sensitive data.

### 5.4 Example bioinformatics analysis project

To illustrate how NeLS is used in daily operation of the help desk of ELIXIR Norway, we include an example. The help desk was contacted by a researcher wanting to analyze 15 RNA-Seq samples from the Atlantic salmon. We decided that the already prepared RNA-Seq workflow in our Galaxy instance at NTNU Trondheim consisting of HISAT2 alignment ^22^, followed by read assignment by featureCounts (subRead package) ^28^ and differential expression analysis in voom ^29^, would be suitable for the initial analysis of the data. Atlantic salmon is not an organism with pre-processed genome and transcriptome readily available, so our help desk first had to create a HISAT2-indexed reference genome from the original Atlantic salmon genome FASTA file and transcriptome GFF file downloaded from SalmoBase ^30^. The initial indexing (using HISAT2 indexing with 1.5TB of memory) was done by the ELIXIR Norway staff at NMBU Ås responsible for SalmoBase. This step only needs to be performed once for any reference genome, and can be reused for other users targeting the same organism. The indexed reference was made available for selection in the workflow by ELIXIR Norway staff at NTNU. To run the workflow, the NTNU help desk created a shared project in NeLS Storage Level I layer, and shared the project with the researcher. They in turn uploaded the raw sequencing data to the shared project in NeLS (then becoming available to the responsible person in the help desk), who ran the RNA-Seq workflow in the Galaxy environment. The workflow made use of the dataset collection feature in Galaxy to run alignment and read-assignment on all 15 samples in a single step. Sample group assignments for comparison in differential expression analysis can be defined at the beginning of the workflow, or by adding assignment and comparisons during a re-run of the last step in the workflow (voom analysis). In this way, the user only needs to run the computationally demanding alignment and assignment steps once, but still have the flexibility to change samples assignments and group comparisons in subsequent analysis. In total, four group comparisons were made, reporting differentially expressed genes in each comparison. The total processing and analysis were done with a minimal effort for the user who basically only had to 1) upload the data, 2) define a dataset collection, 3) select the correct organism reference genome (for alignment) and transcriptome (for counting), and 4) define the sample groups assignments and the groups to be compared (differential analysis).

## 6 Unified service toward data-generating platforms

National or other large data-generating platforms, such as the Norwegian Consortium for Sequencing and Personalized Medicine (NorSeq ^ad^) produce user-requested sequencing for multiple purposes. The data are produced on receipt of DNA samples, and these may be of both human and non-human nature, requiring different data handling procedures. The goal is to provide a unified and seamless user experience, in which the user is provided with a resulting dataset in an environment that is suitably equipped with compute resources, relevant analytical tools, reference data, and initial analysis results. All of this should be provided and documented with no action required from the user after the initial agreement. This requires a tight collaboration between the data-generating platform, the national hardware (storage, compute) resources, as well as the ELIXIR help desk facilitating for the user experience in providing relevant tools, workflows, support, and documentation.

Non-sensitive data handling is coordinated through the use of NeLS and its layered architecture. NorSeq staff uploads the generated data to a NeLS project area created by ELIXIR Norway help desk on behalf of the research group ordering the sequencing. After verification of the uploaded data, ELIXIR Norway help desk assists the research group in synchronizing the data also to the Store-Bioinfo services at the Norwegian Infrastructure for Research Data (NIRD), and provides access in different roles to the different members of the research project. Users may then analyze the data utilizing the Galaxy front ends in the NeLS ecosystem, and receive support and training from the ELIXIR Norway help desk.

Sensitive data handling is achieved by utilization of Services for Sensitive Data (TSD) ^19^. NorSeq staff uploads the generated data to a special TSD project that allows for initial data analysis using ELIXIR Norway provided workflows jointly by NorSeq and ELIXIR Norway help desk staff, before the raw and processed data are made fully available to the user’s TSD project.

## 7 Discussion

We have described the NeLS system developed to serve a broad spectrum of bioinformatics users, with focus on Norwegian users and on genomics data. The system supports data storage, sharing, and data analysis in a project-oriented fashion. A strength of the system is that it utilizes a federated identity provider allowing most users to use their institutional login. Furthermore, it integrates storage and compute resources offered by the generic einfrastructure Sigma2, set-up and funded to support users across all research fields in Norway. This avoids duplication of effort and caters for a more harmonized policy with respect to allocations of compute and storage between life science and other fields. An additional strength of the system is that it has interfaces adapted to both advanced users through a programmatic interface (API) and SSH, and to less computer-savvy users through a web portal. This allows different categories of users to work efficiently with the system, and to collaborate through joint projects. The system has been in production since 2015, and has been adapted according to user feedback accumulated over a series of workshops.

The system has been designed to use existing open-source solutions whenever possible. We believe this strategy produces a system that is easier to maintain and therefore more sustainable. NeLS has been developed in an iterative fashion with short agile development cycles facilitating adaptation to changing needs.

Until now, we have had one instance of NeLS running at the University of Bergen, linked with five instances of Galaxy, one at each of the partner institutions in ELIXIR Norway. For the future, we are investigating a more dynamic approach launching Galaxy instances on demand.

NeLS itself does not provide the level of security required for handling sensitive data. To support such projects, NeLS is linked with the TSD (Services for Sensitive Data) platform in Oslo ^19^. NeLS and TSD are integrated, allowing transfer of data and workflows between the systems, making for more resource-efficient support of both types of projects benefiting both their operation and their users.

NeLS uses the national Federated Electronic Identity provider (FEiDE) linking all Norwegian universities. The technology used is the same as that used for the ELIXIR Authentication and Authorisation Infrastructure (AAI) ^15^. It is therefore possible to extend NeLS to also support ELIXIR AAI identity provision. NeLS has so far been designed and resourced for supporting Norwegian projects, and new policies – and ideally also new funding mechanisms – would be needed to extend the scope beyond Norwegian projects.

The NeLS system can be used as an example of how to set up a flexible and relatively light-weight system providing bioinformatics projects with data storage, sharing, and analysis. The NeLS source code is available on GitHub and can helpin the building of similar projects elsewhere, although adaptations must be expected, for example to integrate with storage and compute resources.

The modularity of NeLS allows its parts to be reused in other contexts. An example is the integration of NeLS with the SEEK platform ^2, 3^, where users can link data sets in NeLS with their metadata in SEEK. Future work may include functionality for allowing users to export annotated data from NeLS (optionally integrating linked metadata from SEEK) into public data repositories such as ArrayExpress ^31^ and PRIDE ^32^.

## 8 Conclusions

The NeLS system is in production and serves as an important platform for the operation of ELIXIR Norway and its help desk for users in molecular life sciences. The system will therefore be maintained and supported in the foreseeable future. We benefit from sharing experiences with other similar projects within and beyond ELIXIR, through the wide adoption of Galaxy across many ELIXIR nodes.

## Data availability

All data underlying the results are available as part of the article and no additional source data are required.

## Software availability

Bio.Tools ^33^ ID: *NeLS* ( https://bio.tools/nels)

RRID: SCR_016301

NeLS is available at https://nels.bioinfo.no, without extra registration for all Norwegian academic users (via FEiDE ^ae^), and with registration upon request for all other users.

The source code of the core NeLS modules is available at https://github.com/elixir-no-nels/nels-core, under the Apache License 2.0 ^af^.

Archived source code at the time of publication is available here: http://doi.org/10.5281/zenodo.1251639 under an Apache License 2.0 ^34^.

## Use cases

The source code of the module integrating NeLS with the national Service for Sensitive Data (TSD), can be found within the above repository and archive in *elixir-no-nels/nels-core/tsd-proxy*.

The source code of the integration module of META-pipe with NeLS, including Galaxy front end and HPC back end (Stallo supercomputer at UiT), is available under the MIT license ^ag^ at https://gitlab.com/uit-sfb/meta-pipe-galaxy-wrapper, archived in 35.

## Notes

a https://basespace.illumina.com

b https://www.geneious.com

c 14, *pp.* 53–56, 61–64, https://bora.uib.no/bitstream/ handle/1956/10658/thesis.pdf#page=61

d https://www.sigma2.no

e https://wiki.oasis-open.org/security/FrontPage

f https://www.feide.no/introducing-feide

g https://simplesamlphp.org

h https://www.rabbitmq.com

i https://projects.spring.io/spring-security/

j https://tools.ietf.org/html/rfc5849

kServices for Sensitive Data, in Norwegian *Tjenester for Sensitive Data*

l https://zenprojects.github.io/Apache-Authmemcookie-Module/

m https://memcached.org

n https://galaxyproject.org/admin/config/pulsar/

o https://galaxy-uit.bioinfo.no

p https://www.adaptivecomputing.com/products/open-source/torque

q https://www.freebsd.org/

r https://en.wikipedia.org/wiki/ZFS

s https://irods.org/

t https://tools.ietf.org/html/rfc5849

u https://pypi.org/project/tornado/

v https://github.com/joestump/python-oauth2

w https://digitallifenorway.org/

x https://filezilla-project.org/

y https://www.norseq.org

z licebase.org

aa https://galaxy-uit.bioinfo.no

abServices for Sensitive Data, in Norwegian *Tjenester for Sensitive Data*

ac https://neic.no/tryggve2/

ad https://www.norseq.org

ae https://www.feide.no/introducing-feide

af https://www.apache.org/licenses/LICENSE-2.0.html

ag https://opensource.org/licenses/MIT
