# Supplementary material for: Norwegian e-Infrastructure for Life Sciences (NeLS)
Source: F1000Res. 2018 Jun 29;7:ELIXIR-968. [Version 1] doi: 10.12688/f1000research.15119.1 (PMC6137412; doi:10.12688/f1000research.15119.1)
Supplement: Peer review history

# Peer review: Referee response for version 1

**DOI:** 10.5256/f1000research.16472.r35602

**Published:** 2018-09-13

**Authors:** Wolfgang Mueller

**Affiliations:** 1 Heidelberg Institute of Theoretical Studies gGmbH, Heidelberg, Germany

I am a member of ELIXIR, and as member of the FAIRDOM project, I am heavily involved with the SEEK system whose link to NeLS is hinted inside the paper. However, I have been only losely involved with that, so I was deemed to be an appropriate reviewer. In order to make things crystal clear, I hereby make my status visible.

 In my view, this paper has diverse functions:

- It provides an architectural view on how a comparatively lightweight combination of a variety of pre-existing tools can yield a powerful national research infrastructure.
- It provides a short justification of many architectural decisions.
- It describes main steps in an ordinary NeLS project.
- It provides a description of typical use cases and a reference of workflows that can be run by users.

I find the paper clear and readable.

 I second the major remark by Olivier Collin.

 I was also surprised to find a whole paper about an infra structure that does not reference the FAIR principles explicitly. I think they could provide some point of reference regarding the handling of metadata, identifiers, licenses.

 However, it is only a matter of making existing links to FAIR more explicit.  

 One suggestion: Please adjust the numbering in the drawings with the numbering of subsections in the paper. This would make things much easier.

 Bullet point 2.3 (c) references subsection 2.4, shouldn't this be 2.8, the public API? Please clarify.

I have read this submission. I believe that I have an appropriate level of expertise to confirm that it is of an acceptable scientific standard.

# Peer review: Referee response for version 1

**DOI:** 10.5256/f1000research.16472.r35600

**Published:** 2018-07-16

**Authors:** Olivier Collin

**Affiliations:** 1 Univ Rennes, Inria, CNRS, IRISA F-35000, Rennes, France

The authors describe the architecture of NeLS, the Norwegian e-Infrastructure for Life Sciences. Use cases provide an overview on how the NeLS infrastructure operates. The infrastructure can deal with sensitive and non-sensitive data.

 The microservices oriented architecture allowing several universities to propose a national service is well explicited. The integration with data-generating platforms will help users to manage efficiently their research processes.

 The article is well written and provides a good overview of the infrastructure.

 The description of the use case improves the understanding of the operations.

 Additional informations and source code are available on a GitHub repository. 

**Comments**

*Major remark :*

 The data management could be more explicitely described in the article.

 The data analysis workflow is well described from the data analysis point of view but the usage of SEEK in the NeLS infrastructure is only briefly mentioned. Some additional description on SEEK usage and interest for NeLS users could improve the article.

 In the Figure 1 : there is a mention of "data curation into structured storage". This data curation is not explained in the text and this raises some questions. Is this data curation using SEEK ?

*Minor remarks :*

 In the abstract, it is stated that "NeLS is also integrated with the SEEK platform in order to store large data files produced by experiments described in SEEK". This should be rephrased since it gives the impression that the SEEK platform is used to store large data files.

 In the second chapter of the introduction, the flow of ideas is not clear. A comparison is made between BaseSpace, Geneious and SEEK before mentioning GenomeSpace. Why compare BaseSpace, Geneious and SEEK that are tools that do not really compare  since the first two are focused on data analysis and the last on data management? Maybe there could be some rephrasing needed (this could also help to improve the data management focus mentioned in my first comment).

 Concerning BaseSpace and Geneious, it could be less judgemental to say that their service is chargeable instead of expensive. And to say that their platform is private.

 It could be interesting to describe briefly why the GenomeSpace setup could not be adapted.

 In the data reproducibility chapter some reference genomes are made available on the five Galaxy instances. Is it possible to describe how this is achieved and how everything is synced?

 Some quantitative data about the number of users are needed in order to better estimate the community covered.

 In the abstract there is a mention of a "project-oriented fashion". This expression is strange to me. Maybe replacing fashion by way or method or mode?

*Typo *:

 The headlines are numbered and sometimes there is a dot after the number and sometimes not. And they are not numbered in the templates if I am not mistaken.

  In 2.1 Authentication

  whether the user is who he claims to be  instead of whether the user is who they

 Page 9 col 1 line 29 : e-infrastructure

 Page 9 col 2 line 15 : some strange font glitches in the pdf file (but not online)

 Page 9 col 2 line 17 : can help in instead of helpin

I have read this submission. I believe that I have an appropriate level of expertise to confirm that it is of an acceptable scientific standard.
